# Examining 21st century skills in BYOD schools: From programs to practice

**DOI:** 10.1007/s35834-024-00425-w

**Published:** 2024-07-09

**Authors:** Maria-Luisa Schmitz, Tessa Consoli, Chiara Antonietti, Alberto Cattaneo, Philipp Gonon, Dominik Petko

**Affiliations:** 1 Institute of Education, University of Zurich (UZH), Kantonsschulstrasse 3, 8001 Zürich, Switzerland; 2 Swiss Federal University for Vocational Education and Training (SFUVET), Lugano and Berne, Switzerland

**Keywords:** Bring-your-own-device, 21st-century skills, One-to-one program, Upper secondary schools, Bring-Your-Own-Device, 21st-century Skills, One-to-One-Programm, Sekundarstufe II

## Abstract

Bring-your-own-device (BYOD) is a common strategy to increase technology integration in schools and give learners more responsibility in using digital devices for educational purposes. In particular, learners are expected to develop domain-general 21st-century skills when using their personal devices. Although there is no consensus regarding a comprehensive framework of 21st-century skills, most conceptual models incorporate aspects such as collaboration, communication, creativity, and critical thinking—so-called 4C competences—as well as self-direction and the use of digital technologies for learning, among other aspects. The importance of these competencies has been stressed in general and vocational education. To study the relationship between BYOD approaches and self-reported 21st-century skills, we conducted a survey of *N* = 8265 upper-secondary students from 100 schools in Switzerland. Using multilevel linear modeling, we compared the self-reported 21st-century skills of students with or without personal digital devices for learning in general and vocational education tracks. Our results indicate that learners reported higher levels of 21st-century skills by indicating that they brought their own devices to school, especially with regard to creativity, self-direction, and the use of technology for learning. However, an official BYOD program was no significant predictor of students’ self-reported skills. The interactions between an official BYOD program and actual BYOD practice in class were not significant. Further, self-reported levels of 21st-century skills seemed to be higher in general than in vocational education. The study indicates that a BYOD concept alone does correspond to students’ 21st-century skills but actual BYOD practices do.

## Abstract (de)

Bring-Your-Own-Device (BYOD) ist eine gängige Strategie, um Technologieintegration in Schulen zu fördern und den Lernenden mehr Verantwortung beim Lernen mit digitalen Geräten zu übertragen. Es wird von den Lernenden erwartet, dass sie bei der Nutzung ihrer persönlichen Geräte fächerübergreifende 21st-century Skills entwickeln. Zwar gibt es keinen Konsens über vollständiges Modell für 21st-century Skills, allerdings umfassen die meisten Modelle, neben anderen Aspekten, Aspekte wie Kollaboration, Kommunikation, Kreativität, kritisches Denken – den so genannten 4K-Kompetenzen – auch Selbststeuerung und die Nutzung digitaler Technologien für das Lernen. Die Bedeutung dieser Kompetenzen wurde sowohl in der allgemeinen als auch in der Berufsbildung betont. Um die Beziehung zwischen BYOD und den selbstberichteten 21st-century Skills zu untersuchen, führten wir eine Umfrage unter N = 8265 Schülerinnen und Schülern der Sekundarstufe II aus 100 Schulen in der Schweiz durch. Mittels Mehrebenenanalyse wurden die selbstberichteten 21st-century Skills von Schülerinnen und Schülern mit und ohne persönliche digitale Geräte für das Lernen in allgemeinen und beruflichen Bildungsgängen verglichen. Die Ergebnisse zeigen, dass die Lernenden ein höheres Niveau an 21st-century Skills berichten, wenn sie angaben, ihre eigenen Geräte in die Schule mitzubringen, insbesondere bei der Kreativität, Selbststeuerung und der Nutzung von Technologien zum Lernen. Ein offizielles BYOD-Programm hatte war kein signifikanter Prädiktor für die von den Lernenden selbst angegebenen Fähigkeiten. Die Interaktionseffekte zwischen einem offiziellen BYOD-Programm und der tatsächlichen BYOD-Praxis im Unterricht waren nicht signifikant. Darüber hinaus scheinen die selbst eingeschätzten 21st-century Skills in der Allgemeinbildung höher zu sein als in der beruflichen Bildung. Die Studie deutet darauf hin, dass ein BYOD-Konzept allein nicht mit den 21st-century Skills der Lernenden korreliert, die tatsächliche BYOD-Praxis jedoch schon.

## Introduction

In education, a bring-your-own-device (BYOD) program mandates that students bring their personal devices to school to use during lessons (Kay and Schellenberg 2019). By contrast, in one-to-one (OTO) laptop programs, the school provides students with digital devices. These two programs differ in four important aspects. First, in a BYOD program, students select and buy digital technologies themselves, which are often laptops but may also include tablets or, in rare cases, even smartphones, whereas in an OTO program, the school chooses the device. Second, in a BYOD program, students install applications and can personalize their devices. In an OTO program, software applications and settings are selected by the school. Third, since students have to afford the devices themselves in a BYOD program, the quality of the devices differs in contrast to an OTO program, where all students are provided with the same device. Fourth, access to digital technologies may be different for the two programs. Whereas students in a BYOD program can always use their devices, not all students in OTO programs might be allowed to use it outside of class, and teachers could have more influence over students’ access to the devices. Based on these theoretically discussed differences it could be expected that schools with OTO programs may provide even but not the highest quality of devices for all students since it might prevent students from having and using their own devices of higher quality (Kay and Schellenberg 2019). By contrast, BYOD programs deal with the problem of equity and access (Adhikari et al. 2017a) leading to a diverse quality of personal digital devices (Fincher 2016; Hopkins et al. 2013). However, Gabriel et al. (2022) noted that many of the devices brought by the students may be more up-to-date than the computers provided by the school.

Compared to OTO programs, BYOD programs seem to have many advantages: they are less costly for schools and might therefore be more popular (Kay and Schellenberg 2019; Keane and Keane 2022; Ottestad and Gudmundsdottir 2018). Students are more familiar with their own devices than with digital technologies provided by the school (Fincher 2016; Janssen and Phillipson 2015). Consistently, Hopkins et al. (2013) showed that students feel confident using their own devices and rarely need help. Since students can use their own devices inside and outside of the class in a BYOD program, they can take more ownership of their learning, engagement, motivation, and technical skills, which might increase (Adhikari et al. 2017a, b; Kay and Schellenberg 2019; Malloy 2019; Parsons and Adhikari 2016). Therefore, it is not surprising that teachers and students appreciate the different devices of the students in the classroom, despite the challenge of dealing with more technical diversity (Fincher 2016; Hopkins et al. 2013). As almost all students in higher grades bring their own devices to school, it is an obvious choice to use these devices as tools for learning (Ott et al. 2018). This is also mirrored by the many European policies discussing the topic of BYOD (Ottestad and Gudmundsdottir 2018). However, there has been little research in the field of BYOD compared to OTO programs, especially in upper secondary schools (Fincher 2016; Kay and Schellenberg 2019; Ross 2013).

### BYOD programs in schools

The few available qualitative studies on BYOD in upper secondary schools have mainly emphasized students’ outcomes and how BYOD is used for learning activities. One case study of students from three upper secondary schools with either a BYOD or an OTO program investigated the purposes for which information and communication technology (ICT) was used. Qualitative content analysis revealed that the students in BYOD programs used digital technologies daily in class mainly to support the writing process, peer support, digital documentation, and information storage (Olofsson et al. 2018). Furthermore, Alirezabeigi et al. (2020) found that in BYOD schools, even traditional learning activities, such as reading and writing, are transformed, and tasks that are administered using personal devices gain a different status beyond isolated units in class. Further, the central role of learning tasks in a BYOD school influences and changes the whole school organization (Alirezabeigi et al. 2020). By contrast, Selwyn et al. (2017) observed no fundamental changes caused by a BYOD approach in their case study. They indicated that the hierarchical nature of the lessons was preserved while implementing a BYOD approach. Further, even the traditional approach was strengthened, as teachers had the opportunity to monitor their students better, and students rarely moved beyond the content presented by the teacher (Selwyn et al. 2017).

Regarding students’ outcomes, qualitative evidence from teacher interviews reveals that the digital literacy skills of students are promoted by a BYOD approach (Adhikari et al. 2017a). Since the BYOD program has led to an extension of learning beyond the formal classroom, students gain higher agency in their learning process and self-efficacy. In terms of learning outcomes, teachers have reported observing an increase in students’ knowledge acquisition and critical thinking. However, teachers have also indicated that students in BYOD classes are more easily distracted by technology (Adhikari et al. 2017a). In another mixed-method case study, students from Texas reported that they were more creative and efficient when they were allowed to use their smartphones during lessons, whereas teachers were concerned about the number of distractions and lack of control in BYOD classes. Furthermore, teachers reported that students’ ability to find ways to use technology for learning purposes was limited without their support (Malloy 2019). A focus group interview study with students from Swedish upper secondary schools indicated that a smartphone is seen as a strong means of supporting the learning process because the students use it for research when they are stuck and thus could reengage in school work. Although students reported that they get distracted by their smartphones, they also reported that the use of mobile phones during lessons can collectively calm down the class, as everybody is sitting quietly while working on tasks on their mobile phones (Ott et al. 2018). Summing up this qualitative evidence, there seem to be benefits and pitfalls to using BYOD in class. Whereas students mainly reported benefits, teachers seemed to be more skeptical.

A few survey studies have shed light on BYOD practices on a larger scale. In New Zealand, Hopkins et al. (2013) revealed that students believed a BYOD approach would facilitate their learning and support them in improving their grades. In another study, Parsons and Adhikari (2016) employed longitudinal quantitative and qualitative data to confirm that students perceived higher agency in their learning process after the implementation of BYOD. They claimed to be more productive in class, communicate better with teachers and classmates, enjoy learning more, and improve their learning outcomes. Furthermore, the students’ self-reported technical skills increased over time (Parsons and Adhikari 2016). In summary, most research on BYOD programs in upper secondary schools shows the positive effects of BYOD programs on students’ agency in the learning process, their learning outcomes, and digital skills. The diversity of findings concerning BYOD’s impact also stems from the varied comparison conditions used for evaluation. While certain studies contrast BYOD initiatives with unspecified non-BYOD scenarios, a more robust approach would entail comparing BYOD with OTO programs. Alternatively, assessing personal technology use in educational settings could involve comparing schools with official BYOD programs against those without such provisions. With regard to measures or proxies of student learning outcomes and academic performance, the evidence is still scarce. While studies on OTO programs have reported mainly positive outcomes depending on the type of use (Dunleavy and Heinecke 2008; Fleischer 2012; Harper and Milman 2016), it is unclear whether BYOD programs outperform OTO programs. However, meta-analyses on mobile learning show higher effect sizes for handheld devices in self-directed and project-oriented learning settings (Sung et al. 2016) and the use of personal devices has been found to be especially beneficial for language learning (Chen et al. 2020). For other subjects, the evidence is still limited and does not differentiate between OTO or BYOD programs (e.g. Garzón and Lampropoulos 2023; Güler et al. 2022). As BYOD transfers the responsibility for their digital devices to the students, it stands to reason whether this corresponds with a transfer towards self-directed learning and the development of related skills. So far, BYOD research lacks a model that systematically summarizes these skills. As research on the general effects of mobile learning has shown that effects are higher for programs with a longer duration and more intensive use, future research needs to distinguish between the effects of an official BYOD program and actual BYOD practices in class on students’ skills. Instead of comparing schools with and without official BYOD programs, the wide-spread availability of personal digital devices among students might enable BYOD practices in schools without an official BYOD approach.

### BYOD programs and students’ 21st century skills

One aspect that could systematically summarize students’ abilities that might be affected by BYOD programs is 21st-century skills. These skills are often seen as domain-general key skills that are crucial to meeting the demands of education, work, and business in the 21st century, extending beyond traditional curricular knowledge and routine problem-solving. There are many different definitions for these types 21st-century skills (for recent frameworks and overviews, see Chalkiadaki 2018; Finegold and Notabartolo 2010; González-Pérez and Ramírez-Montoya 2022; Voogt and Roblin 2010). In contrast to similar frameworks in the field of learning strategies, critical thinking or problem solving, the concept of 21st-century skills is deeply rooted in assumptions regarding the changing skill demands in work and society. As machines are increasingly taking over routine tasks, students require skills that go beyond what computers are able to do and they need to be able to work with computers productively and responsibly (van Laar et al. 2017). A very common categorization of skills in this framework is to differentiate between cognitive (e.g., critical thinking), intrapersonal (e.g., self-direction), interpersonal (e.g., collaboration and communication), and technical skills (e.g., using technology for learning; Geisinger 2016). Although there is no consensus on a specific set of competencies that could be defined as 21st-century skills, most systematic reviews overlap in labeling transversal skills as collaboration, communication, creativity, and critical thinking, as well as self-direction and the use of technology for learning purposes as 21st-century skills in an educational context (e.g., Rychen and Salganik 2003; OECD 2018). Apart from the lack of consensus on a specific list of skills, research on 21st-century skills has many other research gaps. A review by van Laar et al. (2020) found that the determinants of 21st-century skills are rarely studied and are limited to personality and psychological factors. Hence, it could be interesting to investigate whether contextual factors, such as an official BYOD program and the actual BYOD practice in class correspond with collaboration, communication, creativity, critical thinking (known as the 4C skills; www.p21.org), in addition to self-direction, and using technology for learning (Bray et al. 2020).

According to Bray et al. (2020), *Collaboration* can be described as the ability to work on projects with others or solve problems together. It also means working effectively and respectfully with classmates to achieve a common goal while assuming joint responsibility for the fulfillment of tasks. In contrast, *communication* is characterized as organizing thoughts, data, and findings and sharing them effectively through digital technologies. There is little evidence that the implementation of a BYOD program might have positive effects on collaboration and communication skills. In Olofsson et al.’s (2018) study, students reported using their devices on a regular basis for peer support activities. According to Parsons and Adhikari (2016), students in their study indicated that they communicated better with teachers and peers after the implementation of BYOD, which could be due to the fact that personal devices are more frequently used for peer support activities.

According to Bray et al. (2020), *creativity* is the generation of solutions to complex problems based on the analysis and synthesis of information and the presentation of results in new and original ways. In Malloy’s (2019) study, students reported an increase in their creativity when they were allowed to use their own digital devices in class. Indeed, the literature review by Tang et al. (2022) shows that digital technologies can improve students’ creativity. 

*Critical thinking* encompasses analyzing complex problems, investigating questions without definitive answers, evaluating information sources, and using adequate evidence to draw conclusions (Bray et al. 2020; Lu and Xie 2023). Adhikari et al. (2017a) found that teachers in BYOD classes offered students more innovative learning activities, greater flexibility, and agency, and therefore the learners’ critical thinking abilities increased.

*Self-direction* means that students are responsible for their own learning by identifying topics to pursue and learning processes. Students are also able to monitor and review their own work and respond to feedback (Bray et al. 2020). Many studies indicate that a BYOD approach helps students take more ownership of their learning (Adhikari et al. 2017a, b; Kay and Schellenberg 2019; Malloy 2019; Parsons and Adhikari 2016), which could promote students’ self-directed learning skills. However, Selwyn et al. (2017) reported that students in BYOD classes rarely moved beyond the content presented by the teacher.

Lastly, *the use of technology for learning* is described as creating and managing products to learn using suitable technology. Although most studies showed that a BYOD approach could help students to efficiently use technology and improve their learning outcomes (Adhikari et al. 2017a, b; Ott et al. 2018; Parsons and Adhikari 2016), Malloy (2019) found that students’ ability to use technology for learning was limited without teacher guidance in BYOD classes. In summary, there is little qualitative evidence that a BYOD approach has mostly positive effects on the six key skills of the 21st-century frameworks, and findings from large-scale assessment studies are completely lacking.

### Research question

As past research has shown, the effects of One-to-One initiatives and learning with mobile devices are not only primarily dependent on the provision of technology but on the duration, intensity and purpose of technology use that is empowered in these programs. BYOD programs in particular, were found to support a change toward more self-directed learning which might in turn foster 21st century skills. Today, where literally every student in secondary education possesses a personal digital device that can be used for learning, the question of whether BYOD makes a difference needs to be reframed, however (Ott et al. 2018). For example, the case study by Olofsson et al. (2018) revealed that practices described in an official BYOD concept might differ from actual classroom practices. Although students needed to bring their own devices they had to do pen and paper exercises in class (Olofsson et al. 2018). Therefore, this study endeavors to answer the question of whether the presence of an official BYOD program in a school still yields discernible differences compared to schools without such initiatives. Moreover, it is warranted to examine which role the actual BYOD practices during a lesson play next to the official BYOD program. Furthermore, the advantages and disadvantages of BYOD practices compared to OTO practices were only discussed on a theoretical basis (Fincher 2016; Janssen and Phillipson 2015; Kay and Schellenberg 2019; Keane and Keane 2022). Hence, this study aims to investigate both practices in schools. Based on the gaps in previous research, this study aims to answer the following exploratory research questions:

1. Does an official BYOD program correspond with students’ self-reported 21st-century skills?
2. Do actual BYOD or OTO practices in class correlate to students’ self-reported 21st-century skills?

For the first research question, we expect that students in schools with an official BYOD program will report higher levels of 21st-century skills than students in schools without an official BYOD program. We assume that an official BYOD concept should support actual BYOD practices: students in a school with an official BYOD concept who use their personal devices in class should report significantly higher skill levels than students using their own personal devices in a school without an official BYOD concept.

Regarding the second research question, we hypothesize that students who report using their own digital devices in class tend to rate their own 21st-century skills significantly better than students in classes that do not use a personal device. Although BYOD use is likely in schools with an official BYOD program, BYOD use can also happen independently. Furthermore, we postulate that students who indicate using a personal device provided by the school (OTO) would report significantly higher levels of skills than those who do not use a personal device, as previous research has also shown positive effects of OTO programs on students’ skills (Kay and Schellenberg 2019). We have no clear prediction for the differences between students who are confronted with a BYOD approach and those in an OTO program. Based on previous findings, BYOD and OTO approaches both have specific strengths and weaknesses. On the one hand, the flexibility of a BYOD approach that allows students to use their digital devices inside and outside of the school leads to more ownership of learning, which increases motivation and engagement and can have positive effects on students’ self-reported skills (Adhikari et al. 2017a, b; Kay and Schellenberg 2019; Parsons and Adhikari 2016). On the other hand, students in a BYOD approach might have problems with equity and access; in particular, those who have access only to low-quality digital devices might report lower skill levels than students in an OTO program (see Adhikari et al. 2017b; Fincher 2016; Kay and Schellenberg 2019). Since we assume that students in the general baccalaureate track, who spend their entire school week using these devices, will have greater benefits from a BYOD approach than students in the vocational track, who only spend one to two days per week in school and the other days on the job, we also include the school type as a control variable.

## Methods

### Participants and procedure

The first survey wave for the canton of Zurich was from September 20 to November 8, 2020, followed by the second survey wave for all other cantons in Switzerland from May 1 to August 1, 2022. The first wave also served as a pretest for the national study. We contacted all Swiss upper-secondary schools to invite students who were enrolled in the penultimate year of upper-secondary school to participate in an online survey. A total of 8915 students from 108 schools completed our online questionnaire. We also surveyed school principals (*N* = 225). Matching student data with principals’ responses led to a final sample of 8265 students from 100 schools. This sample represents 19% of the upper secondary schools and 20% of the total student population in Switzerland in the penultimate year of their studies.

Overall, 47.3% of the students indicated male as their gender, 49.2% identified as female, and 3.5% chose the option “other” for their gender. For the language region, 81.3% reported going to a school in the German-speaking region, 10.5% of the students were located in the francophone region, and 8.2% of the students were enrolled in a school in the Italian-speaking region. Overall, 31.5% of the students were enrolled in general education, 53.9% were vocational education students, and 14.5% were in schools that combined general and vocational education. Regarding the official BYOD program, 56.6% of students were in schools with a school-wide BYOD program, 32.1% were in a school with a BYOD program for some classes, and 11.3% were in a school with no BYOD program. However, 78.4% of the students reported bringing their own devices to class (BYOD), 5.4% of the students indicated that they used devices in class that were provided by the schools (OTO), and 16.2% did not use personal devices in school. Table 1 is a cross-table that shows the number of students in the BYOD programs and practices.

**Table 1:** Cross-table BYOD program and practice

		BYOD program			
		School-wide (%)	Some classes (%)	None (%)	Total (%)
BYOD practice	None	2.9	9.6	3.7	16.2
	BYOD	51.9	20.0	6.5	78.4
	OTO	1.8	2.4	1.1	5.4
	Total	56.6	32.1	11.3	100

*N* = 8265

### Measurements

#### Official BYOD concept

Regarding the official BYOD concept, 225 school principals were asked whether they had a BYOD program at their schools (“Does your school implement a bring-your-own-device policy (i.e., each student is required to bring a personal digital device to class)?”). Three answer options were provided in the survey: 1 (*Yes*), 2 (*In some classes*), and 3 (*No*). The responses of the school principals were added to the student dataset. In 56 schools, more than one principal per school responded. In 18 schools, the answer options chosen by the school principals differed. If there was a discrepancy between the responses of the principals from the same school, the response category chosen by the greatest number of respondents was selected (which applied to eight schools). The only exceptions were six schools, in which even though the majority of principals selected the “No” response option, the response option was coded as “In some classes” because at least one principal was found to have selected the “Yes” or “In some classes” option for the school. In four schools, an equal number of principals selected the “Yes” and “In some classes” response options; thus, for the student dataset of these four schools, the response was coded as “In some classes.” These discrepancies might be attributed to the fact that school leaders from the same school might answer this question with regard to different organizational units within the school. As the questions in the questionnaire were clearly worded with regard to the whole school, it might be reasonable to assume that a majority of the respondents understood them correctly.

#### BYOD practices in class

Regarding the actual BYOD practice in class, students were asked to indicate if students in their class used a personal device during the lessons (i.e., “Do the students in your class use a personal computer, a notebook, or tablet at school?”). The answer options were 1 *(No)*, 2 *(Yes, personal devices),* and 3 *(Yes, personal devices provided by the school)*. The second answer option can be categorized as a BYOD practice, whereas the third answer option is an OTO practice.

#### Students’ 21st-century skills

For students’ 21st-century skills, we used Bray et al.’s (2020) scale. Students were asked to rate themselves on six key skills, which were measured with three items each: collaboration (e.g., “Work in pairs or small groups to complete a task together”; Cronbach’s α = 0.83; McDonald’s ω = 0.83), communication (e.g., “Prepare and deliver an oral presentation to the teacher or others”; Cronbach’s α = 0.81; McDonald’s ω = 0.81), creativity (e.g., “Create something new that can help you express your ideas”; Cronbach’s α = 0.82; McDonald’s ω = 0.82), self-direction (e.g., “Assess the quality of your work before it is completed”; Cronbach’s α = 0.76; McDonald’s ω = 0.77), critical thinking (e.g., “Analyze different arguments, perspectives, or solutions to a problem”; Cronbach’s α = 0.79; McDonald’s ω = 0.80), and using technology for learning (e.g., “Use technology to keep track of your work on assignments”; Cronbach’s α = 0.81; McDonald’s ω = 0.81). The answer options ranged from 1 (*Not good at all*) to 5 (*Very good*). A confirmatory factor analysis was conducted using Jamovi (2.3.0) to confirm the categorization into the six different key skills. The model showed a good fit without any modification (chi^2^ (120) = 2907; *p* < 0.001; TLI = 0.951; CFI = 0.962; RMSEA = 0.053; SRMR = 0.035), given that the following was considered a good fit for the different indices: > 0.95 for the TLI and CFI, and < 0.08 for the RSMEA and SRMR (Brown 2015; Hu and Bentler 1999).

### Statistical analyses

#### Descriptive statistics

For the mean scales of the six key skills (collaboration, communication, creativity, self-direction, critical thinking, and using technology for learning), the means and standard deviations were calculated. Since a survey with a full population was not achieved, sampling weights were applied to achieve unbiased population estimates with regard to school responses, school type, and language region (Kish and Frankel 1974; Meinck 2015). The following weighting formula was used:

$$\begin{aligned}&\text{Weight}=a(N\,\text{of students who are enrolled in the school}\\&\qquad/N\,\text{of respondents in the school})\\
&\times b(N\text{ of students in }\mathrm{a\ }\text{language region}/N\text{ of respondents in }\mathrm{a\ }\text{language region})\\
&\times c(N \mathrm{}\:\text{ of students enrolled in }\mathrm{a\ }\text{school type}/N\text{ of respondents in }\mathrm{a\ }\text{school type})\\&
\times (\text{mean}(a\times b\times c))\end{aligned}$$

For multilevel structural equation modeling, we used unweighted data to avoid distorted results (see Gelman 2007; Winship and Radbill 1994).

#### Multilevel linear modeling

Since students were nested in 100 schools (clusters), we considered multilevel linear modeling (MLM) analyses to investigate the effects of different BYOD aspects and school types on students’ key skills. First, for each of the six 21st-century skills, a single-level model was conducted with the school as a predictor. This single-level model was compared through the analysis of variance to a fully unconditional model (null model) with a random intercept to test whether an MLM approach was appropriate for the nested data. The intra-class correlation (ICC) was also calculated. Then, for each of the six key skills, a random intercept MLM analysis was performed with the BYOD concept, BYOD practice, the interaction of the concept and practice, and school type as predictors (model 1). Again, the fit of these models was compared with the null models’ fit. The analyses were performed using Jamovi (2.3.0) and the package lmertest in R (4.2.2). Moreover, post hoc tests with Bonferroni correction were performed to examine the effects of the predictors in more detail.

## Results

### Descriptive statistics

Table 2 shows that the highest mean was reported for collaboration, whereas the lowest means were reported for creativity and critical thinking. However, the differences are small, with students judging their abilities as positive on average. More details on the means and standard deviations across different BYOD practices and programs can be found in the appendix.

**Table 2:** Descriptive statistics for students’ 21st-century skills

Variable	Mean	SD
Collaboration	3.60	0.92
Communication	3.54	0.96
Creativity	3.47	0.89
Self-direction	3.58	0.83
Critical Thinking	3.47	0.86
Technology for Learning	3.55	0.94

Means and standard deviations (*SD*) are weighted

### Multilevel linear modeling

Table 3 shows that the ICCs for the null models range from 0.01 to 0.05, indicating that between 1 and 5% of the variance in students’ key skills can be attributed to the differences between schools. These ICCs can be considered rather small. However, even with a low ICC, the Type I error rate may be much higher than the conventionally used alpha level. Thus, MLM approaches are preferred as long as the sample size requirements are met (see Huang 2018). As a rule of thumb says that at least 30 clusters (schools) are required for MLM analysis (Hox 1998), for our sample with 100 clusters (schools), it is advisable to consider the nested structure of the data. In line with this, we found that the null models fit the data significantly better than the single-level models. Furthermore, all models 1 have a significantly better fit than the null models, except Model 1 for creativity.

**Table 3:** Results of Multilevel Linear Modeling Analyses Predicting Students’ 21st-Century Skills

	Collaboration		Communication		Creativity		Self-direction		Critical Thinking		Technology for Learning	
	Null model	Model 1	Null model	Model 1	Null model	Model 1	Null model	Model 1	Null model	Model 1	Null model	Model 1
*Fixed*												
Intercept	3.67 (0.02)***	3.76 (0.06)***	3.61 (0.02)***	3.62 (0.7)***	3.50 (0.01)***	3.39 (0.06)***	3.60 (0.02)***	3.53 (0.06)***	3.50 (0.02)***	3.47 (0.06)***	3.60 (0.03)***	3.54 (0.07)***
BYOD concept Partially − Yes	–	−0.06 (0.07)	–	0.03 (0.07)	–	0.05 (0.07)	–	0.04 (0.06)	–	0.06 (0.07)	–	0.01 (0.08)
BYOD concept No − Yes	–	−0.05 (0.08)	–	0.02 (0.09)	–	0.05 (0.08)	–	0.04 (0.08)	–	0.07 (0.08)	–	0.09 (0.10)
School type VET − General	–	−0.19 (0.03)***	–	−0.21 (0.03)***	–	−0.04 (0.03)	–	−0.12 (0.03)***	–	−0.18 (0.03)***	–	−0.28 (0.04)***
School type Mixed − General	–	−0.03 (0.05)	–	0.02 (0.04)	–	0.01 (0.04)	–	0.01 (0.04)	–	0.00 (0.04)	–	0.03 (0.06)
BYOD practice BYOD − None	–	0.01 (0.06)	–	0.10 (0.06)	–	0.12 (0.06)*	–	0.16 (0.05)**	–	0.13 (0.06)*	–	0.22 (0.06)***
BYOD practice OTO − None	–	−0.18 (0.09)*	–	−0.08 (0.09)	–	0.00 (0.09)	–	0.03 (0.08)	–	0.04 (0.09)	–	0.08 (0.10)
*Interactions*												
Partially − Yes * BYOD − None	–	0.09 (0.07)	–	−0.01 (0.08)	–	−0.04 (0.07)	–	−0.05 (0.07)	–	−0.07 (0.07)	–	−0.01 (0.08)
No − Yes * BYOD − None	–	0.04 (0.09)	–	−0.09 (0.10)	–	−0.02 (0.09)	–	−0.11 (0.08)	–	−0.09 (0.09)	–	−0.15 (0.10)
Partially − Yes * OTO − None	–	0.25 (0.12)*	–	0.22 (0.12)	–	0.11 (0.11)	–	0.06 (0.11)	–	0.08 (0.11)	–	0.06 (0.12)
No − Yes * OTO − None	–	0.30 (0.14)*	–	0.11 (0.15)	–	0.10 (0.14)	–	0.14 (0.13)	–	−0.02 (0.13)	–	0.19 (0.15)
*Random*												
Intercept	0.02 (0.13)	0.01 (0.08)	0.02(0.14)	0.01 (0.08)	0.00 (0.6)	0.00 (0.06)	0.01 (0.11)	0.01 (0.08)	0.02 (0.13)	0.01 (0.09)	0.04 (0.21)	0.22 (0.15)
*Model fit*												
ICC	0.02	0.01	0.02	0.01	0.01	0.00	0.02	0.01	0.02	0.01	0.05	0.03
Log likelihood	−10,786.56	−10,761.97	−11,166.91	−11,138.34	−10,562.64	−10,553.90	−9889.80	−9866.62	−10,318.71	−10,296.45	−11,017.21	−10,974.15
df	3	13	3	13	3	13	3	13	3	13	3	13
*p*	≤ 0.001	≤ 0.001	≤ 0.001	≤ 0.001	≤ 0.001	0.0064	≤ 0.001	≤ 0.001	≤ 0.001	≤ 0.001	≤ 0.001	≤ 0.001
Reference	Single level model	Null model	Single level model	Null model	Single level model	Null model	Single level model	Null model	Single level model	Null model	Single level model	Null model

Estimates and standard errors from the multivariate random intercept model for fixed effects, variances, and standard deviation for the random effects * significant at the 0.05 level; ** significant at the 0.01 level; *** significant at the 0.001 level

Table 3 shows that students who indicated that students used their own devices in class (BYOD practice) reported significantly higher creativity, self-direction, critical thinking, and technology for learning skills than students who indicated that they did not use a personal device in school. However, the post hoc test showed no significant difference between these two groups in critical thinking (difference = −0.08, *p* = 0.058). Regarding the post hoc tests for using technology for learning, students who indicated that they did not use a personal device in class rated their skills significantly lower than students who indicated that they used a digital device provided by the school (OTO practice) (difference = −0.16, *p* < 0.001). For collaboration, students who used devices provided by the school in class reported significantly lower skills than students who did not use personal devices. This difference was not significant in a post hoc test (difference = 0.00, *p* = 1.00). For all other key skills, the difference between being a student in an OTO program and not using personal devices was not significant. There was no significant difference between the BYOD and OTO programs with regard to students’ 21st-century skills.

Moreover, the official BYOD concept reported by the school leaders had no significant relationship with students’ 21st-century skills. Regarding the interaction effects between an official BYOD concept reported by the school leaders and the actual BYOD practice reported by students, no significant effects were found in the MLM analyses. There were only two exceptions. First, students in schools with an official BYOD concept in some classes who used devices provided by the school reported significantly higher collaboration skills than students in schools with an official BYOD concept who did not use their personal devices. Second, students in schools with no official BYOD concepts who used devices provided by the school reported significantly higher collaboration skills than students in schools with an official BYOD concept who did not use personal devices. These effects did not remain significant in the post hoc tests.

Regarding the post hoc tests, some significant interaction effects were observed for self-direction and using technology for learning. Students in schools with a BYOD concept for some classes who did not use a personal device reported significantly lower self-direction skills than students in schools with a school-wide BYOD concept who indicated using their own devices in class (difference = −0.13, *p* = 0.046). Students in schools with a BYOD concept for some classes who did not use personal devices in class reported significantly lower skills than students in schools with a BYOD program for some classes who used their own devices (difference = −0.20, *p* < 0.001). Furthermore, students in schools with a BYOD concept for some classes who indicated that they did not use a personal device reported significantly lower skills than students who used their own devices during lessons in schools with a BYOD concept for all classes (difference = −0.20, *p* = 0.006). Moreover, students in schools with a schoolwide BYOD concept who did not use personal devices reported significantly lower skills in using technology for learning than students in schools with a schoolwide BYOD concept who used their own devices in class (difference = −0.22, *p* = 0.015). However, no significant differences can be found between students who used their own devices in schools with a schoolwide BYOD concept, in schools with a BYOD concept for some classes, or in schools without a BYOD concept.

Table 3 also illustrates that for each of the six 21st-century skills except creativity, school type was a significant predictor. Students from general education reported significantly higher 21st-century skills than students from vocational education. Moreover, post hoc analyses showed that students in vocational education schools rated their skills in collaboration (difference = −0.16, *p* = 0.002), communication (difference = −0.23, *p* < 0.001), self-direction (difference = −0.13, *p* = 0.008), critical thinking (difference = −0.18, *p* < 0.001), and use of technology for learning (difference = −0.31, *p* < 0.001) significantly lower than students from schools that combine vocational and general education.

## Discussion

### Summary of the results

Since only a minority of students in our sample reported never using their own personal devices in class, Ott et al.’s (2018) statement that BYOD affects almost all schools seems to be a reality in Switzerland. For the first research question, contrary to our expectations, we found that there was no significant difference in students’ 21st-century skills between learners in schools with an official BYOD program and those without any program. Although unbalanced group sizes and small variances could have influenced the results by decreasing the statistical power of the analysis, it seems that not an official program but the actual BYOD practices in class positively corresponded to students’ self-reported 21st-century skills. In line with this assumption, we found that even the interactions between an official BYOD concept and actual BYOD practices had no significant relation with students’ self-reported 21st-century skills. Contrary to our expectations, students who used their own devices in schools with a schoolwide BYOD concept did not report significantly higher 21st-century skills than students who used their own devices in schools without a BYOD concept. These findings suggest that a BYOD concept has no incremental influence beyond actual BYOD practices on self-reported skills. However, since we did not analyze the specifics of the BYOD programs among the participating schools, this finding merely indicates that there are no discernible differences in results between schools where BYOD is mandatory and those where it is not. This doesn’t imply the ineffectiveness of BYOD programs overall. It is reasonable to assume that certain BYOD initiatives may promote effective practices. This has not been analyzed in this study, however. Regarding the second research question, our study confirms previous mostly qualitative findings that BYOD practices have positive relations with students’ self-reported creativity, self-direction, and the use of technology for learning skills (Adhikari et al. 2017a, b; Kay and Schellenberg 2019; Ott et al. 2018; Parsons and Adhikari 2016). These results challenge the findings of Selwyn et al. (2017), who indicated that BYOD practices did not support students in moving beyond the content explained by the teacher, and Malloy (2019), who found that students’ ability to use technology for learning was limited in BYOD classes. Not all 21st skills were positively correlated with BYOD practices. In particular, there were no significant relationships present in our analyses and post hoc tests for BYOD practice with self-reported critical thinking, collaboration, and communication. One explanation for this might be the unbalanced sample sizes of the different groups for practices and the limited variance leading to small effects. However, future research is needed to clarify the relationship between BYOD practices and self-reported skills further.

Although previous studies revealed that OTO programs positively correspond with students’ skills (Kay and Schellenberg 2019), we found no significant differences in students’ 21st-century skills between learners practicing OTO and students who indicated that they had neither a BYOD nor an OTO practice in class. The only exception was that, according to the post hoc test, students working with an OTO approach reported higher skill levels in using technology for learning than learners who did not use a personal device. However, these results could have been influenced by unbalanced sample sizes and limited variances. Furthermore, we investigated the differences between OTO and BYOD practices. No significant differences in 21st-century skills were found between these two groups. On the one hand, this finding might be surprising regarding the fact that students using BYOD devices can use their own devices more flexibly and take more ownership of their learning, which leads to higher engagement, motivation, and skill levels than students using OTO devices (Adhikari et al. 2017a, b; Kay and Schellenberg 2019; Malloy 2019; Parsons and Adhikari 2016). On the other hand, in contrast to OTO devices, BYOD devices face the challenges of equity and access (Adhikari et al. 2017b; Fincher 2016; Kay and Schellenberg 2019). Thus, if students have only access to low-quality digital devices, a BYOD approach might also correspond negatively with self-reported skills. Also, this study did not investigate in detail which practices were prevalent in detail and whether they differed. It is very like that effects are not dependent on the type of device but on the type of use. Aside from unequal group sizes and limited variance, the nonsignificant differences between BYOD and OTO device-use in terms of students’ 21st-century skills may also be explained by the fact that the advantages and disadvantages of the two programs balance out and that both approaches can lead to similar practices.

Concerning school type, our study revealed that, except for creativity, students from vocational education reported significantly lower skill levels than those from general education and those from schools combining general and vocational education. This could be attributed to the different curricula of the school types that focus on fostering different skills but also to the frequency of using digital devices at school. Another potential explanation is that students from vocational education could have underestimated their skill levels compared to those from general education.

### Limitations

The first limitation of this study relates to the structure of the data, as the group sizes for students in the different BYOD implementation forms (for the official programs and the practice) are unequal. However, MLM analysis is a robust procedure with regard to unbalanced group sizes (Field 2018). Nevertheless, the unequal group size might result in less precision in the estimates for the smaller group, a greater sensitivity towards outliers in the smaller group and less statistical power for group comparisons. Second, we found very limited variance in our sample, which led to only small effects. The third limitation concerns the measurements of the study. For the BYOD practices, students were asked to indicate whether students in their class use personal devices. This measurement of BYOD practices rather covers the BYOD behavior of others and may or may not reflect the student’s individual behavior. To measure students’ 21st-century skills, we asked them to self-indicate their skill level instead of using objective performance measures. Nevertheless, it may be worthwhile to determine whether implementing a BYOD approach increases student self-efficacy in important skill areas. Fourth, although most frameworks of 21st-century skills encompass the six key skills surveyed in this study (communication, collaboration, creativity, self-direction, critical thinking, and using technology for learning), it is still unclear how many skills should be included in the frameworks. As there is little research on BYOD programs in schools, the interpretation of the correlative results is not supported by a strong theoretical background. Nevertheless, the majority of previous qualitative findings point very consistently to a positive relationship between BYOD practices and self-reported skills. Moreover, this study is an important first step to shed more light on the underresearched BYOD topic. An additional limitation concerns our sample having data from only Swiss upper secondary schools; hence, no inferences can be drawn for other school levels or countries.

### Theoretical and practical implications

Regarding the theoretical implications, future research should investigate whether these findings can be replicated for other school levels and countries. Moreover, it would be interesting to conduct longitudinal studies to examine the effects of following a BYOD approach on students’ skills (see Adhikari et al. 2017a; Parsons and Adhikari 2016). Future studies could also aim to determine whether a BYOD program has a positive impact on students’ skills measured with objective performance tests. As we only differentiated between two different aspects of BYOD—an official BYOD program and actual BYOD practices in class—future research could focus on other aspects, such as the quality of equity and access to a BYOD program or how long the BYOD program has been implemented (Adhikari et al. 2017a, b; Kay and Schellenberg 2019; Malloy 2019; Parsons and Adhikari 2016).

In terms of practical implications, our study provides evidence for policymakers and school leaders that a BYOD approach might be beneficial to promote students’ self-efficacy with regard to creativity, self-direction, and technology for learning skills in upper secondary schools. Having an official BYOD program might be a good first step in supporting students’ self-efficacy in important skills, but what really matters is actual BYOD practices in class. In the case study by Olofsson et al. (2018), students reported that some teachers told them to bring their own laptops to school but gave them just pen-and-paper exercises. Without leveraging the advantages of a BYOD approach, positive correlations with self-efficacy in 21st-century skills rarely occur. In the end, teachers are key players in effectively implementing the BYOD program and enabling students to feel competent in important skill areas.

## Appendix

**Table 4:** Cross-table BYOD program and 21st century skills with means and standard deviations

		21st century skills					
		Collaboration	Communication	Creativity	Self-direction	Critical thinking	Technology for learning
BYOD program	School-wide	3.58 (0.93)	3.54 (0.96)	3.49 (0.87)	3.61 (0.81)	3.50 (0.85)	3.58 (0.91)
	Some classes	3.61 (0.92)	3.55 (0.97)	3.46 (0.92)	3.55 (0.85)	3.44 (0.88)	3.50 (0.98)
	None	3.71 (0.89)	3.55 (0.96)	3.33 (0.89)	3.58 (0.84)	3.50 (0.86)	3.59 (0.91)

Means and standard deviations are weighted. *N* = 8265

**Table 5:** Cross-table BYOD practice and 21st century skills with means and standard deviations

		21st century skills					
		Collaboration	Communication	Creativity	Self-direction	Critical thinking	Technology for learning
BYOD practice	None	3.61 (1.02)	3.56 (1.01)	3.42 (0.98)	3.51 (0.92)	3.46 (0.95)	3.46 (1.01)
	BYOD	3.60 (0.90)	3.54 (0.95)	3.47 (0.87)	3.59 (0.80)	3.46 (0.84)	3.55 (0.92)
	OTO	3.61 (0.91)	3.57 (0.98)	3.53 (0.93)	3.65 (0.87)	3.59 (0.89)	3.70 (0.98)

Means and standard deviations are weighted. *N* = 8265

**Table 6:** Cross-table BYOD program * BYOD practice and 21st century skills with means and standard deviations

		21st century skills					
		Collaboration	Communication	Creativity	Self-direction	Critical thinking	Technology for learning
BYOD program * BYOD practice	School-wide * None	3.54 (1.12)	3.26 (1.18)	3.30 (0.96)	3.39 (1.01)	3.34 (0.96)	3.23 (1.00)
	School-wide * BYOD	3.58 (0.91)	3.56 (0.94)	3.51 (0.86)	3.63 (0.78)	3.52 (0.84)	3.60 (0.90)
	School-wide * OTO	3.42 (0.89)	3.45 (0.97)	3.45 (0.93)	3.52 (0.95)	3.40 (0.92)	3.48 (0.93)
	Some classes * None	3.60 (1.01)	3.62 (0.97)	3.49 (0.99)	3.54 (0.89)	3.48 (0.96)	3.51 (1.02)
	Some classes * BYOD	3.62 (0.89)	3.51 (0.97)	3.42 (0.89)	3.54 (0.83)	3.39 (0.84)	3.47 (0.95)
	Some classes * OTO	3.60 (0.91)	3.63 (0.99)	3.62 (0.92)	3.66 (0.86)	3.66 (0.89)	3.68 (1.02)
	None * None	3.70 (0.98)	3.56 (0.95)	3.24 (0.93)	3.50 (0.94)	3.48 (0.92)	3.43 (0.93)
	None * BYOD	3.63 (0.81)	3.56 (0.97)	3.43 (0.82)	3.56 (0.73)	3.47 (0.82)	3.52 (0.86)
	None * OTO	3.92 (0.83)	3.51 (0.96)	3.32 (0.95)	3.76 (0.81)	3.60 (0.81)	4.10 (0.78)

Means and standard deviations are weighted. *N* = 8265
